# *Trans*-fat labelling information on prepackaged foods and beverages sold in Hong Kong in 2019

**DOI:** 10.1017/S1368980022002464

**Published:** 2022-11-23

**Authors:** Christopher Chi Wai Cheng, Jason HY Wu, Jimmy Chun Yu Louie

**Affiliations:** 1 School of Biological Sciences, Faculty of Science, The University of Hong Kong, 5S-14 Kadoorie Biological Sciences Building, 1 Pokfulam Road, Pokfulam, Hong Kong; 2 Food Policy Division, The George Institute for Global Health, Camperdown, NSW, Australia

**Keywords:** *Trans*-fat, Pre-packaged foods, Hong Kong, Food labelling, Accuracy

## Abstract

**Objective:**

To examine the labelling status of *trans*-fat of pre-packaged foods sold in Hong Kong.

**Design:**

Data from 19 027 items in the 2019 FoodSwitch Hong Kong database were used. Ingredient lists were screened to identify specific (e.g. partially hydrogenated vegetable oil, PHVO) and non-specific *trans*-fat ingredient indicators (e.g. hydrogenated oil). *Trans*-fat content was obtained from the on-pack nutrition labels, which was converted into proportion of total fat (%_total fat_). Descriptive statistics were calculated for *trans*-fat content and the number of specific, non-specific and total *trans*-fat ingredients indicators found on the ingredients lists. Comparisons were made between regions using one-way ANOVA and *χ* ^2^ for continuous and categorical variables, respectively.

**Setting:**

Cross-sectional audit.

**Participants:**

Not applicable.

**Results:**

A total of 729 items (3·8 % of all products) reported to contain industrially produced *trans*-fat, with a median of 0·4 g/100 g or 100 ml (interquartile range (IQR): 0·1–0·6) and 1·2 %_totalfat_ (IQR: 0·6–2·9). ‘Bread and bakery products’ had the highest proportion of items with industrially produced *trans*-fat (18·9 %). ‘Non-alcoholic beverages’ had the highest proportion of products of ‘false negatives’ labelling (e.g. labelled as 0 *trans*-fat but contains PHVO; 59·3 %). The majority of products with *trans*-fat indicator originated from Asia (70 %).

**Conclusions:**

According to the labelling ∼4 % of pre-packaged food and beverages sold in Hong Kong in 2019 contained industrially produced *trans*-fat, and a third of these had *trans*-fat >2 %_total fat_. The ambiguous *trans*-fat labelling in Hong Kong may not effectively assist consumers in identifying products free from industrially produced *trans*-fat.

*Trans* fatty acids are found in industrially produced partially hydrogenated vegetable oils (PHVO), as well as in small quantities in fats from ruminants^(1)^. Compared with non-hydrogenated vegetable oils, PHVOs have increased stability and shelf life, as well as a flavour profile that is more similar as SFA, at a fraction of costs of the latter^(2)^. Hence until recently, PHVOs were a popular choice of ingredient among food industry to manufacture foods with desired textures and flavours at a lower cost^(3)^.

*Trans*-fat, particularly those industrially produced, had been consistently linked to negative health effects^(3)^, including at least in part higher CVD risks^(1,4–7)^. This is because of the harmful effect of *trans*-fat on cholesterol metabolism – simultaneously increasing the level of LDL cholesterol and decreasing the level of HDL cholesterol^(8,9)^. *Trans*-fat intake has also been shown to be positively associated with all-cause mortality, risks of cognitive disorder and inflammation^(1,10,11)^. As a result, government and public health agencies around the world have introduced plans and legislations to reduce the population’s *trans*-fat intake. For example, in May 2018, the WHO proposed the ‘REPLACE *trans*-fat’ action plan, with a goal to remove industrial *trans*-fat ingredients from the global food supply chain by 2023^(12)^. The USA has completely banned the use of PHVO in pre-packaged foods in 2018^(13)^. In 2019, the European Commission imposed a *trans*-fat limit (for industrially produced *trans*-fat only) of ≤ 2 % of total fat (%_total fat_) to in pre-packaged food, with full enforcement starting in 2021^(14,15)^. Many countries around the world, however, still do not have plans in place to eliminate industrial *trans*-fat from the food supply – particularly many Asian and African countries^(16)^.

While food retailers, importers and manufacturers in Hong Kong need to comply with the Nutritional Label Scheme which mandated the labelling of total *trans*-fat (i.e. both industrially produced and ruminant *trans*-fat) on the nutrition information panel^(17)^, the accuracy of such labeling is seldom studied. In May 2021, 68·4 % of cheeses sampled in Hong Kong were found to have inaccurate *trans*-fat labelling^(18)^. Together with ambiguous names used in the ingredients list, such as ‘vegetable fat’ without specifying whether the fat was hydrogenated; or ‘hydrogenated vegetable oil’ without specifying the degree of hydrogenation, it may be difficult for the general public to correctly identify foods containing *trans*-fat by reading food labels. In fact, two recent Brazilian studies suggested that ingredients with ambiguous names, such as ‘vegetable fat’, were found in foods claimed to have no *trans*-fat^(19,20)^. Given Hong Kong imports most of its pre-packaged food supply, the same issue may also be present in Hong Kong.

Therefore, this cross-sectional study aimed to describe the proportion of pre-packaged foods: (1) exceeding the EU industrially produced *trans*-fat limit of ≤ 2 %_total fat_^(15)^; (2) labelled to contain specific and non-specific ingredient(s) indicative of the possible presence of *trans*-fat and (3) labelled as *trans*-fat free but contains specific ingredient(s) indicative of the presence of *trans*-fat. We also examined differences between food categories and region of origin.

## Methods

### Data source and collection

FoodSwitch Hong Kong is a project that collected data of pre-packaged foods available for sale in Hong Kong annually between 2017 and 2019^(21)^. The 2019 version of the FoodSwitch Hong Kong database^(22)^ was used in this study. Details of the data collection protocol were described elsewhere^(21)^. In brief, data were collected from one megastore each, all from affluent areas, of City!Super (selling mainly imported products), Marks and Spencer (selling a wide range of its home brand products originating from the UK), AEON (selling mainly products imported from Japan), as well as Park’nShop and Wellcome (both selling numerous imported and typical local branded products). Altogether these supermarket chains account for more than 70 % of the market share of pre-packaged foods^(23)^. The data were collected by trained research assistants who visited the stores, who took photographs of each product including the nutrition information panel, front-of-package and barcode, using a bespoke smartphone application^(24)^.

### Data entry and pre-processing

For each food and beverage, data recorded in the 2019 FoodSwitch Hong Kong database include the brand name, product name, barcode, as well as content of total energy, protein, total fat, saturated fat, *trans*-fat, total carbohydrate, total sugar and sodium listed. If the *trans*-fat content per serving or package was displayed on the label, it was converted to the *trans*-fat concentration per 100 g or per 100 ml of the food. It should be noted that the nutrition labelling of Hong Kong does not require the separate reporting of industrially produced *v.* ruminant *trans*-fat. The countries of origin were identified using the 3-digit prefix of the Global Trade Item Number standard barcode^(25)^, which identifies the issuance country, as a proxy if that corresponds to a single country, e.g. ‘489’ for Hong Kong. For items which have Global Trade Item Number prefixes that correspond to more than one issuance country, or items with non-Global Trade Item Number-standard barcodes, the on-pack declaration of country of origin was used. The countries of origin were then grouped into four regions, namely Asia, Europe, North America and others (including Australia, New Zealand, South America, the Middle East and Africa)^(22)^.

### Trans-fat ingredient ‘indicators’

*Trans*-fat ingredient ‘indicators’ were ingredient terms used to determine whether food items labelled as containing 0 g *trans*-fat may potentially contain industrially produced *trans*-fat. Specific *trans*-fat ingredient indicators referred to ingredients known to contain industrially produced *trans*-fat, presence of which indicate ‘false negatives’ for products that were labelled as having zero *trans*-fat on the nutritional label. Non-specific *trans*-fat ingredient indicators referred to ingredients that may or may not contain industrially produced *trans*-fat. A full list of specific (*n* 31) and non-specific (*n* 113) *trans*-fat ingredient indicators is provided as Supplemental Table 1. Of note is that ‘vegetable oils/fats’ without specification of hydrogenation level were considered as non-specific *trans*-fat ingredient indicators in foods that are likely to have industrially produced *trans*-fat, including biscuits, cake and pastries, salad dressings, snack foods and chocolates^(26)^.

### Product categorisation and exclusion criteria

Categorisation of food products was based on the food classification system developed by the George Institute for Global Health^(24)^, which categorises products into eighteen major food groups. The food categories ‘alcohols’ (*n* 6), ‘eggs’ (*n* 67), ‘special foods’ (*n* 335), ‘vitamins and supplements’ (*n* 5) and ‘unable to be classified’ (*n* 23) were excluded as they are unlikely to contain *trans*-fat or not considered a major source of *trans*-fat in the local diet, leaving thirteen major food groups for final analysis. If the same item is sold in multiple stores (as identified by the same GTIN barcode), only one entry of that item was included in the database. For the same item in different package sizes (e.g. cola soft drink in 600 ml bottle *v.* 6 × 330 ml cans), they were manually identified by a researcher (CCWC), based on the brand name, product name and nutrition information, and only one entry was included in the data set for statistical analysis. To better align with the EU *trans*-fat limit which refers only to industrially produced *trans*-fat, the remaining items which reported to contain *trans*-fat but have no *trans*-fat ingredient indicator in the ingredients list (*n* 1463), suggesting the *trans*-fat is likely of ruminant origin, were excluded from the relevant analyses.

### Statistical analysis

Data analyses were conducted using SPSS (version 26; IBM Corp.). The median and interquartile range of *trans*-fat content per 100 g of food and as %_total fat_, stratified by food categories were computed. The proportion of *trans*-fat containing items which has an industrially produced *trans*-fat content above the EU limit of ≤2 %_total fat_^(15)^ was also calculated. The number of specific, non-specific and total *trans*-fat ingredients indicators for each item was counted. Results were stratified by major food categories, and region of origin was applicable. Pearson’s *χ* ^2^ was used to test for the differences between the proportion of items with different number of *trans* fat indicator in the ingredients list from different regions. The differences in the mean ± sd *trans*-fat content (%_total fat_) based on the information in nutrition label of items from different regions were tested using one-way ANOVA with Tukey’s correction for multiple comparison. A two-tailed *P* < 0·05 was considered statistically significant.

## Results

**Fig. 1:**
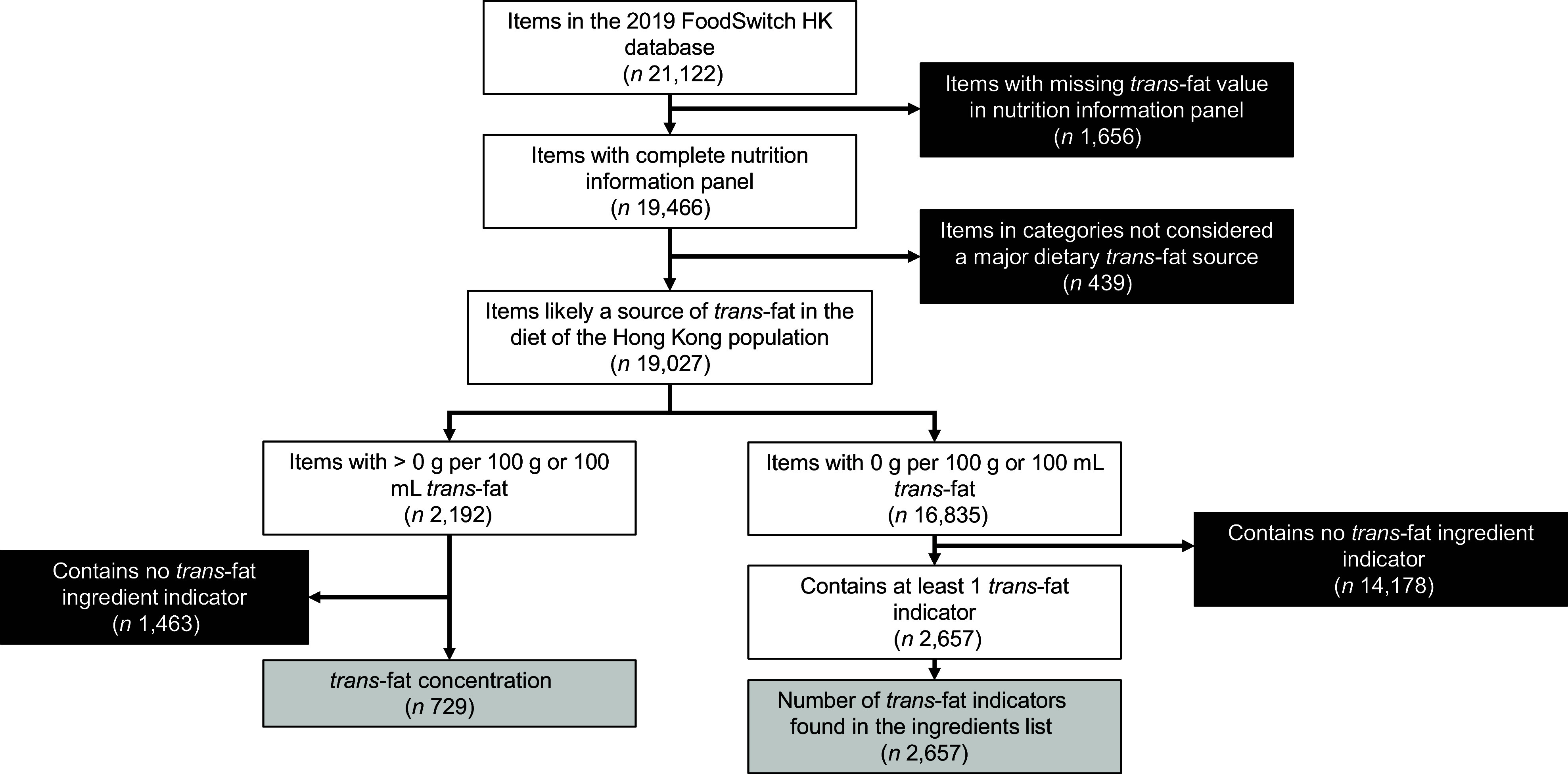
Flow of data preparation and analysis. Black boxes represent exclusion, and grey boxes represent statistical analyses

Of the 21 122 records in the 2019 FoodSwitch Hong Kong database, 1656 products were excluded to have incomplete nutritional information panels without *trans*-fat values, and 439 were excluded due to not being a main source of *trans*-fat in the diet of the Hong Kong population (Fig. 1). Among the remaining 19 027 items, 2192 (11·5 %) reported a *trans*-fat content greater than 0, and 729 of these also contained at least 1 *trans*-fat ingredient indicator in the ingredients list, suggesting the *trans*-fat contained in them was likely to be industrially produced (Table 1). The median *trans*-fat content of these 729 items was 0·3 g/100 g or 100 ml (interquartile range (IQR): 0·1–0·6) and 1·2 %_totalfat_ (IQR: 0·6–2·9). Of these, items in ‘edible oils and oil emulsions’ (median = 1·2, IQR = 0·6–1·4), ‘sauces, dressings, spreads and dips’ (median = 0·6, IQR = 0·3–1·0) and ‘non-alcoholic beverages’ (median = 0·5, IQR = 0·3–0·6) had the highest *trans*-fat concentration (g per 100 g or 100 ml), while ‘dairy’ (median = 3·3, IQR = 2·1–4·4), ‘non-alcoholic beverages’ (median = 3·1, IQR = 1·6–4·7) and ‘meat and meat products’ (median = 2·9, IQR: 2·3–3·6) had the highest proportion of fat as *trans*-fat (%_totalfat_). In contrast, ‘cereal and grain products’ had the lowest median *trans*-fat concentration (median = 0·1, IQR = 0·1–0·3), while ‘fruit and vegetables’ (median = 0·7, IQR = 0·5–1·3) and ‘snack foods’ (median = 0·6, IQR = 0·4–1·3) had the lowest proportion of fat as *trans*-fat. ‘Bread and bakery products’ had highest proportion of items with industrially produced *trans*-fat (18·9 %). ‘Snack foods’ had 9·7 % and ‘confectionery’ 6·6 %. Overall, the EU *trans*-fat limit of ≤ 2 %_totalfat_ was exceeded by 1·3 % of the included products, and ‘bread and bakery products’ (6·8 %) and ‘snack foods’ (1·7 %) had the highest proportion of items exceeding the EU limit.

**Table 1:** *Trans-fat* content reported on nutrition labels of the sampled pre-packaged foods

Food categories	Total *n*	Contains *trans*-fat*, *n*	%	*Trans*-fat content†				Exceeding EU *trans*-fat limit, *n*	%
				Per 100 g or 100 ml (g)		% of total fat (%)			
				Median	IQR	Median	IQR		
Bread and bakery products	1731	328	18·9	0·3	0·2–0·7	1·3	0·7–3·0	117	6·8
Cereal and grain products	2704	31	1·1	0·1	0·1–0·3	0·9	0·6–2·3	9	0·3
Confectionery	1657	109	6·6	0·3	0·2–0·5	0·9	0·6–2·1	27	1·6
Convenience foods	1078	23	2·1	0·2	0·1–0·4	1·9	1·4–2·8	11	1·0
Dairy	1609	19	1·2	0·3	0·1–0·9	3·3	2·1–4·4	15	0·9
Edible oils and oil emulsions	523	15	2·9	1·2	0·6–1·4	2·0	1·3–5·2	6	1·1
Fish and fish products	612	2	0·3	0·1	0·1–0·1	1·0	–	0	0·0
Fruit and vegetables	2374	9	0·4	0·3	0·2–0·4	0·7	0·5–1·3	1	0·0
Meat and meat products	673	9	1·3	0·2	0·1–1·0	2·9	2·3–3·6	8	1·2
Non-alcoholic beverages	2322	32	1·4	0·5	0·3–0·6	3·1	1·6–4·7	22	0·9
Sauces, dressings, spreads and dips	2239	53	2·4	0·6	0·3–1·0	1·4	0·9–2·6	20	0·9
Snack foods	1006	98	9·7	0·2	0·1–0·4	0·6	0·4–1·3	17	1·7
Sugars, honey and related products	499	1	0·2	–	–	–	–	1	0·2
Total	19 027	729	3·8	0·3	0·1–0·6	1·2	0·6–2·9	254	1·3

* *Trans*-fat that was likely to be industrially produced, based on the presence of *trans*-fat ingredient indicators in the ingredients list.

† Only for items reported to have > 0 g/100 g or 100 ml *trans*-fat with 1 or more *trans*-fat ingredient indicators in the ingredients list.

**Fig. 2:**
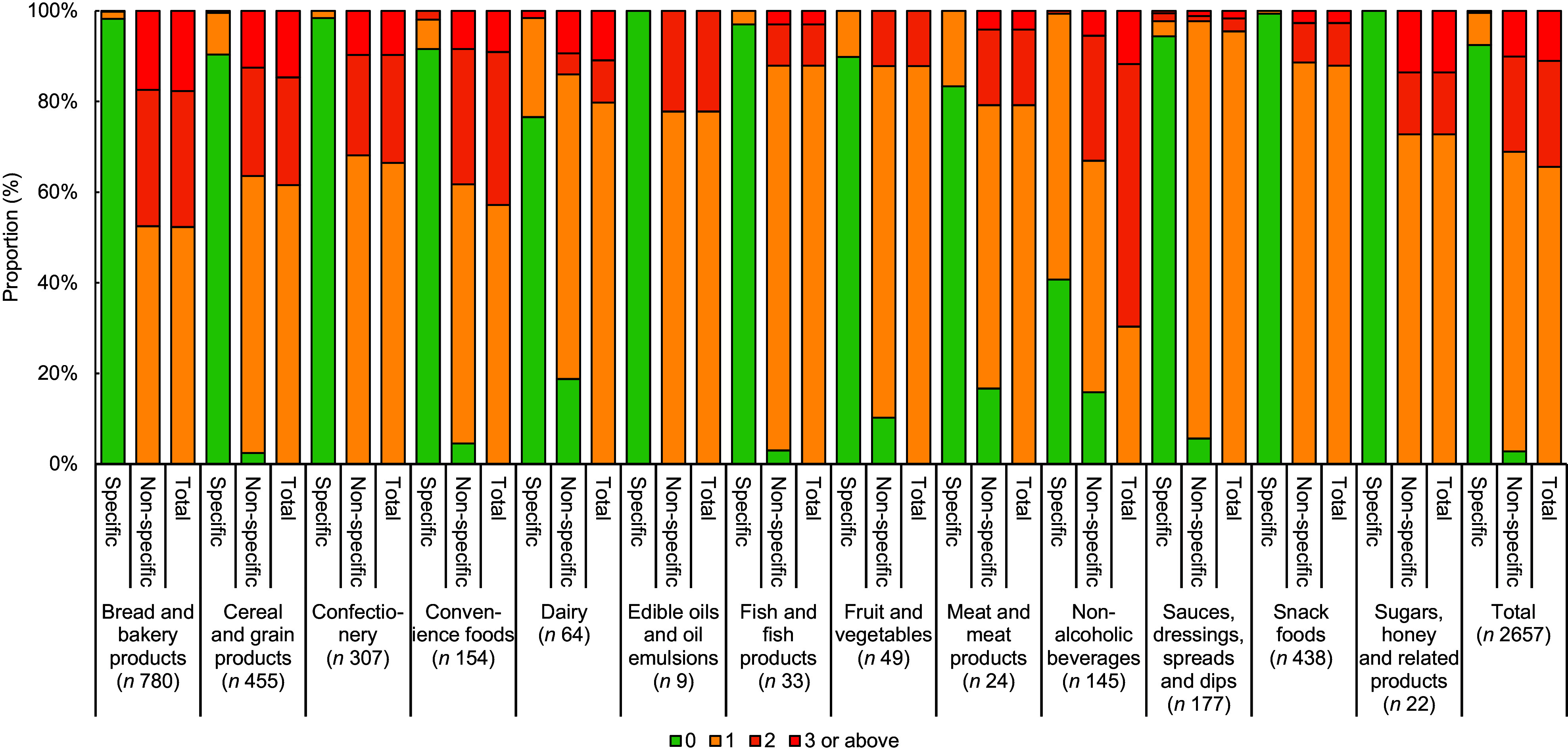
Number of specific, non-specific and total *trans*-fat ingredients indicators in the ingredients list of pre-packaged items labelled as having 0 g *trans*-fat and contain at least 1 *trans*-fat ingredients indicator *(n* 2657), stratified by category

Specific and non-specific *trans*-fat ingredient indicators were found in 234 (1·2 %) and 3298 (17·3 %) of the 19 027 included items, respectively. For items labelled as having 0 g *trans*-fat (*n* 16 835), 14 173 (84·2 %) were found to have no specific or non-specific *trans*-fat ingredients indicators on the ingredient list, suggesting the *trans*-fat labelling was correct. Of the remaining 2657 items, 200 (7·5 %) were found to have at least 1 specific *trans*-fat indicator in the ingredients list (Fig. 2). ‘Non-alcoholic beverages’ was found to have the highest proportion of products of ‘false negatives’ (59·3 %), followed by ‘dairy’ (23·4 %) and ‘meat and meat products’ (16·7 %).

In terms of products with non-specific *trans*-fat ingredient indicators, the majority (97·2 %) of the 2657 items had at least one of these indicators in their ingredients list, indicating they were ‘possible false negatives’. All items in ‘confectionery’, ‘edible oils and oil emulsions’, ‘snack foods’ and ‘sugars, honey and related products’ contained at least one non-specific *trans*-fat ingredient indicators. ‘Bread and bakery products’ (17·7 %), ‘cereal and cereal grain products’ (14·8 %) and ‘sugar, honey and related products’ had the highest proportion of items with three or more total *trans*-fat ingredients indicator.

**Fig. 3:**
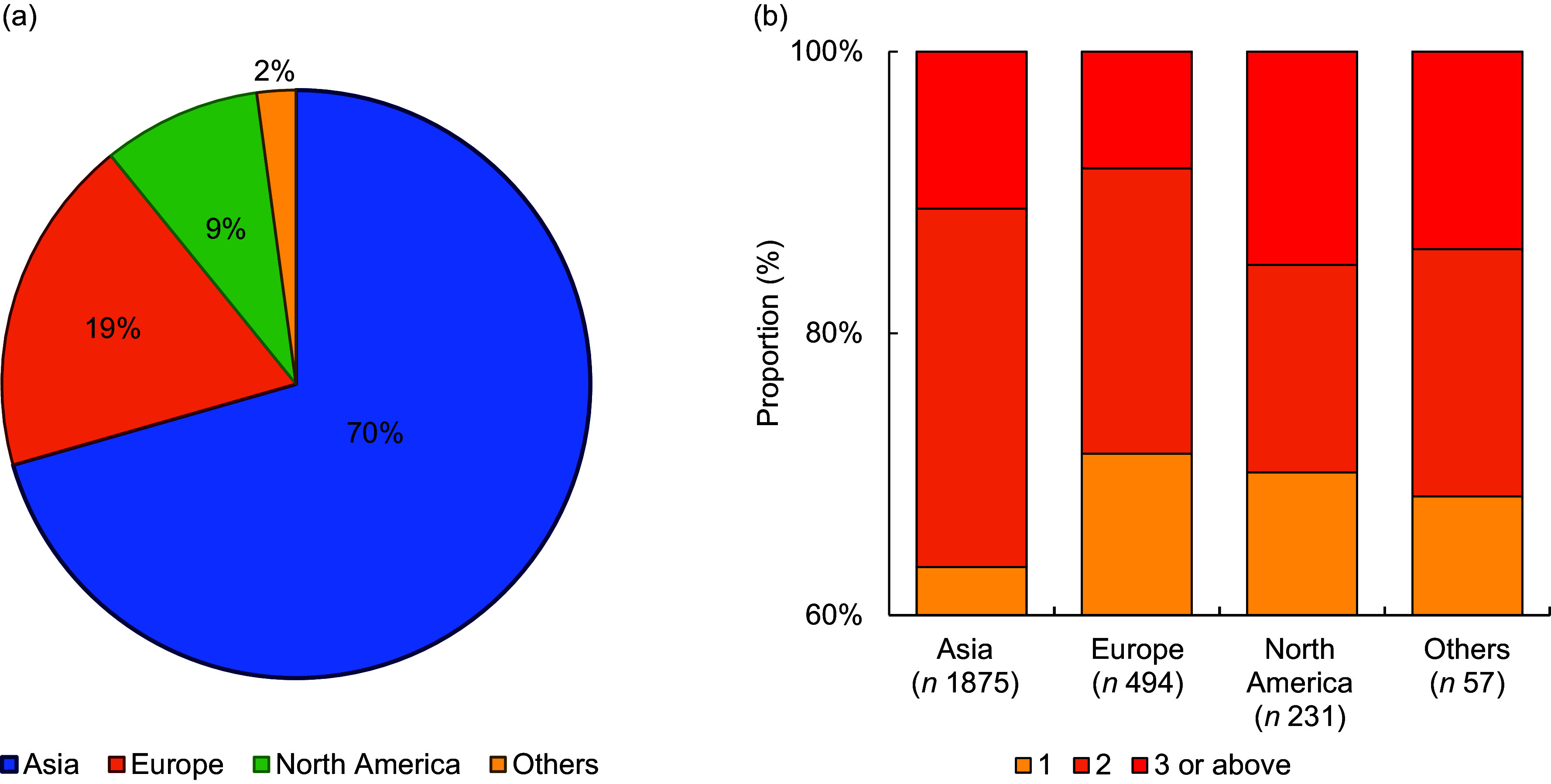
(a) The proportion of pre-packaged items labelled as having 0 g *trans*-fat and contain at least 1 *trans*-fat ingredients indicator from different regions of origin; (b) the number of total *trans*-fat ingredients indicators in the ingredients list of pre-packaged items labelled as having 0 g *trans*-fat and contain at least 1 *trans*-fat ingredients indicator *(n* 2657), stratified by region of origin. Differences were statistically significant for the following pairs: Asia *v.* Europe, *P* = 0·036; Asia *v.* North America, *P* = 0·003

**Fig. 4:**
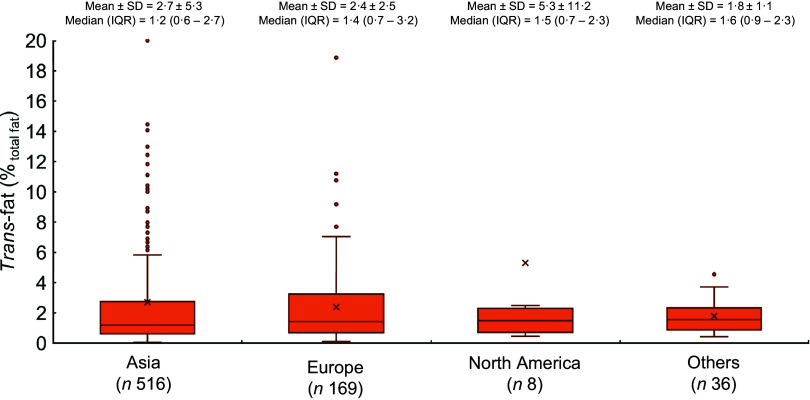
Boxplot of the trans-fat content (as % of total fat) of pre-packaged items reporting to have > 0 g *trans*-fat and have at least one *trans*-fat ingredient indicator in the ingredients list (total *n* 729), stratified by region of origin. For better layout of the figure, nine outliers for Asia (y = 53·9, 53·7, 45·7, 41·3, 28·6, 28·2, 26·4, 25·0, 21·0) and one outlier for North America (y = 33·0) were not displayed. No significant differences between regions were observed (*P*_ANOVA_ = 0·247)

Most products with *trans*-fat ingredient indicators originated from Asia (70 %; Fig. 3(a)), which also tended to have more *trans*-fat ingredients indicators in their ingredients list compared with items from Europe (*P* = 0·036) or North America (*P* = 0·003), and no statistically significant difference was observed between other region pairs (Fig. 3(b)). When comparing the *trans*-fat content (as %_totalfat_) between items labelled as having > 0 g *trans*-fat from different regions (Fig. 4), no significant differences between regions were observed (*P*_ANOVA_ = 0·247).

## Discussion

In our audit, we found that 3·8 % of the pre-packaged foods in Hong Kong likely contained industrially produced *trans*-fats according to the nutrition labels, and around 7·5 % of the sampled items labelled as having zero *trans*-fat had likely mislabelled their true *trans*-fat content. We also found that 1·3 % of the sampled items exceeded the EU industrially produced *trans*-fat limit^(15)^. The use of non-specific *trans*-fat ingredient indictors also appeared widespread, which could potentially mislead the general public to make a wrong decision in food purchasing selection.

Our findings suggest that there were two major issues with the *trans*-fat labelling requirements in Hong Kong. First, we found that among the 16 835 items labelled as zero *trans*-fat, 200 indeed contained specific *trans*-fat ingredients indicators (suggesting that they are ‘false-negatives’), and 2581 items contained non-specific *trans*-fat ingredients indictors in their ingredients lists. This is likely due to the lenient tolerance limit of the ‘*trans*-fat free’ claim in Hong Kong, which is set at < 0·3 g/100 g or 100 ml of food. Consumers may be misled by this lenient limit to believe items possibly containing industrially produced *trans*-fat as ‘*trans*-fat free’, which is counter-intuitive to the general health advice of lowering industrially produced *trans*-fat intake. A more stringent ‘*trans*-fat free’ limit of industrially produced *trans*-fatty acids (e.g. < 0·1 g/100 g or 100 ml) should be imposed to minimise the potential to mislead consumers. Second, we found that the use of non-specific *trans*-fat ingredient indicators in the ingredients lists is widespread. For example, fat or oil ingredients were often labelled ambiguously regarding their hydrogenation level, making it difficult for consumers to identify potential sources of industrially produced *trans*-fat by reading the ingredients lists, a strategy often suggested by health professionals in educating consumers^(27,28)^. Similar issues regarding ambiguity of *trans*-fat ingredients labelling were reported by researchers from Brazil^(19,20)^, Australia^(29)^ and Saudi Arabia^(28)^.

An interesting observation in our study is that non-alcoholic beverages have the highest level of industrially produced *trans*-fat. This is likely a result of the food categorisation system used in the current study, where beverage mixes such as 3-in-1 coffees (containing non-dairy creamer) and coffee/tea creamers are included under the food group ‘non-alcoholic beverages’. With these items (*n* 32) excluded, all remaining items were found to either be labelled as zero *trans*-fat or have no *trans*-fat ingredient indicator in the ingredients list. Similar issues were observed for ‘dairy’, which included ‘dairy alternatives’ (e.g. soya cheese and imitation cream) which may contain industrially produced *trans*-fat.

We also found that most items found to contain *trans*-fat ingredients indicator were from Asian countries. This is likely the nature of the market in Hong Kong, where people in general prefer products imported from other Asian countries for similar taste preferences, as well as lower costs. Products imported from other parts of the world are generally more often found in high-end, niche retail outlets, thereby limiting their presence in the market.

Among items labelled as having >0 g *trans*-fat, those from the ‘Other’ region were found to have the highest *trans*-fat content (as %_totalfat_). Nonetheless, the data used in this study were collected in 2019, before the enforcement of the new EU limit of industrially produced *trans*-fat of ≤ 2 %_total fat_ in April 2021^(14)^, which should be taken account when considering this finding. There is currently no such limit in Hong Kong^(30)^.

Food reformulation is often considered an effective way to remove *trans*-fat from the food supply^(31)^, which has been quite successful in the USA^(32,33)^. To reduce *trans*-fat in pre-packaged foods, ideally manufacturers should either replace them with *cis*-unsaturated fatty acids which can maximise health benefits by simultaneously reducing *trans*-fat and increasing unsaturated fat intake^(33–35)^ or replace PHVO with vegetable oil solidified using interesterification^(36)^, instead of replacing *trans*-fat with saturated fats^(33)^. However, since Hong Kong imports most of its pre-packaged food supply^(37)^, food reformulation is not something that the Hong Kong government has direct regulatory control.

Given the intake of *trans*-fat has been consistently shown to be associated with the risk of CVD and dyslipidaemia^(38,39)^, our findings are concerning as they suggest Hong Kong is quite far from achieving the goal to remove industrial *trans*-fat in the food supply set out in the WHO ‘REPLACE *trans*-fat’ action plan^(12)^. To facilitate the achievement of this goal, the Hong Kong government has recently passed an amendment to the relevant regulation that prohibits from 1 December 2023 the import and sales of any foods, fats and oils that contain partially hydrogenated oils^(40)^, which is a great step forward. Meanwhile, as discussed above, the Hong Kong government should also mandate the use of standardised terminology for oils and fats in food labels, as well as impose a more stringent ‘*trans*-fat free’ limit^(20)^. The government should continue its effort in regularly examining the *trans*-fat content of the local food supply, with a focus on items more likely to contain *trans*-fat such as bread and bakery products and edible oils.

There are several strengths of our study. First, we utilised a pre-packaged food database that represents ∼70 % market share of grocery, meaning we should have covered the major dietary sources of *trans*-fat of the local Hong Kong population. Second, we have examined not only the nutrition information panel for *trans*-fat concentration but also the terms for specific and non-specific *trans*-fat ingredients indicators to identify potential ‘false negative’ similar to the work of Ricardo *et al*.^(20)^

However, we caution the readers to some limitations to our study. First, selection biases may have been introduced as products available only in supermarkets not sampled or stores such as convenience stores were not covered, although as explained above we do not believe this is critical when considering the main dietary sources of *trans*-fat of the local population. Second, although attempts have been made to differentiate ruminant *v.* industrially produced *trans*-fat based on the presence/absence of *trans*-fat ingredient indicators in most products, we were unable to do so for products containing a mix of the two types of *trans*-fat (e.g. cakes that use both dairy cream and palm oil). Since the EU *trans*-fat limit refer to industrially produced *trans*-fat only^(15)^, we may have over-estimated the proportion of products exceeding the limit. Third, our audit found several items having > 60 % of total fat as *trans*-fat, which appears abnormal. However, upon further investigation, these were items reported to contain ∼0·5 g *trans*-fat/100 g with a low total fat content (e.g. 0·9 g/100 g), which may be due to errors in labelling, or that the manufacturers/importers chose to report a value that is at or above the cut-off of *trans*-fat free definition (< 0·3 g/100 g) in a bid to stay within the tolerance limit and avoid breaching the food labelling regulations. Last, the *trans*-fat concentration was obtained from the nutrition information panel instead of chemical analysis. However, conducting chemical analysis on all sampled items is logistically and financially unfeasible. Instead, future studies should focus on assessing the *trans*-fat concentration in products with ‘false negative’ *trans*-fat labelling using gas chromatography-mass spectrometry (GC-MS).

## Conclusions

Around 4 % of pre-packaged food and beverages sold in Hong Kong in 2019 still likely contained industrially produced *trans*-fat, and about a third of these foods exceeded the EU limit of industrially produced *trans*-fat. The current *trans*-fat labelling requirements in Hong Kong are ambiguous and may not be effective in assisting consumers in identifying products free from *trans*-fat. Surveillance of *trans*-fat concentration in pre-packaged food and beverages likely to contain industrially produced *trans*-fat should continue until the removal of *trans*-fat from the food supply is mandated in Hong Kong.

## Supplementary material

For supplementary material accompanying this paper visit https://doi.org/10.1017/S1368980022002464. click here to view supplementary material
