# Supplementary material for: Trans-fat labelling information on prepackaged foods and beverages sold in Hong Kong in 2019
Source: Public Health Nutr. 2022 Nov 23;26(2):315–22. doi: 10.1017/S1368980022002464 (PMC13076084; doi:10.1017/S1368980022002464)
Supplement: Supplementary file 1 [file S1368980022002464sup001.docx]

**Supplementary Table 1 –** Ingredients terms searched

| **Type of indicator** | **Type of ingredient** | **Ingredient terms** |
| --- | --- | --- |
| **Specific** | Partially hydrogenated oils | - partially hardened oil - partially hydrogenated canola oil - partially hydrogenated coconut oil - partially hydrogenated lard - partially hydrogenated oil - partially hydrogenated palm fat - partially hydrogenated palm kernel oil - partially hydrogenated palm oil - partially hydrogenated soybean oil - partially hydrogenated soybean oil with tbhq - partially hydrogenated vegetable fat from palm - partially hydrogenated vegetable oil - partly hardened coconut oil - partly hardened palm kernel oil - partly hydrogenated coconut oil - partly hydrogenated palm kernel oil - refined partially hydrogenated vegetable oils and fats |
|  | Non-dairy creamers | - beverage whitener - cappuccino base creamer - coffee creamer - non dairy creamer - non dairy vegetable cream powder - nondairy creamer - non-dairy creamer - non-dairy creamer concentrate - non-dairy creamer sachet - non dairy whitener - vegetable cream - vegetable cream powder - vegetable cream powder 1 - vegetable cream powder 2 |
| **Non-specific** | Palm oil | - certified sustainable palm oil - coconut and palm kernel oils - contain palm oil - edible palm oil - expeller pressed organic palm fruit oil - fractionated palm kernel oil - fractionated palm oil - organic palm fat - organic palm fruit oil - organic palm oil - organic sustainable palm oil - organic wild hearts of palm - palm & rapeseed blended oil - palm and colza vegetable oils and fats - palm based vegetable fat - palm fat - palm fractions oil - palm fruit oil - palm grease - palm kernel fat - palm kernel oil - palm kernel olein - palm oil - palm oil base - palm oil carotene - palm oil powder - palm oil seasoning powder - palm oleic oil - palm olein - palm olein oil - palm stearin - palm stearin ester oil - palm stearin oil - palm vegetable oil - palm-based vegetable fat - palmist oil - powdered palm oil - rbd palm oil - refined palm oil - refined palm olein - segregated sustainable palm oil - sustainable palm fruit oil - sustainable palm oil - sustainably sourced palm oil - vegetable palm oil |
|  | Hardened / hydrogenated oil or creamer (level of hydrogenated not specified) | - creamer - creamer powder - creaming powder - dairy creamer - edible hydrogenated palm oil powder - evaporated creamer - hardened coconut oil - hardened vegetable fat - hydrogenated canola oil - hydrogenated coconut oil - hydrogenated cottonseed oil - hydrogenated fat - hydrogenated palm fat - hydrogenated palm kernel fat - hydrogenated palm kernel oil - hydrogenated palm kernel olein - hydrogenated palm oil - hydrogenated rapeseed oil - hydrogenated soybean oil - hydrogenated vegetable fat - hydrogenated vegetable oil - interesterified and hydrogenated soybean oil - palm hardened oil - palm kernel hydrogenated oil - refined fully hydrogenated coconut oil - refined hydrogenated coconut oil - refined hydrogenated palm kernel oil - refined hydrogenated palm oil - sweetened condensed creamer |
|  | Margarine | - breakout margarine - fat spread margarine - margarine - vegetable margarine |
|  | Shortening | - cocoa mass shortening - organic palm shortening - organic shortening - palm oil shortening - palm shortening - pork shortening - powdered shortening - shortening - shortening oil - shortening powder - vegetable oil shortening - vegetable shortening - vegetarian shortening |
|  | Processed oil | - edible processed fat - edible processed oil and fat - edible processed oils - edible refined and processed oils - edible refined processed fat and oil - edible refined processed fats - edible refined processed oil - fat oil processed products - flavour processed oil and fat - oil processed food - processed almond oil - processed edible oil - processed edible oil and fat - processed oil - processed oil and fat - processed oil product - processed rapeseed oil - processed rice bran oil |
|  | Modified oil | - edible refined modified oil - modified oil - modified palm kernel oil - modified palm oil |
|  | *Vegetable oils/fats | - animal & vegetable fat - animal & vegetable fat & oil - blended vegetable oil - browned in vegetable oil - contains vegetable fats and oils - edible vegetable fat - edible vegetable fat & oil - edible vegetable oil - edible vegetable oil and fat - high oleic vegetable oil - omega 3 or 6 mixed vegetable oil powder - organic vegetable oil - powder vegetable oil - powdered vegetable oil - pure vegetable oil - refined edible vegetable fat - refined vegetable oil - seasoned vegetable oil - vegetable fat - vegetable fat & oil - vegetable oil - vegetable oil & fat - vegetable oil and fats refined - vegetable oil blend - vegetable oil coated - vegetable oil food - vegetable oil of coconut - vegetable oil or fat - vegetable oil powder - vegetable oil product - vegetable oils in varying proportions - virgin vegetable oils - w3/6 mixed vegetable oil powder |

*Only considered a non-specific *trans*-fat ingredient indicator in the following food categories: biscuits, cakes and pastries, snack foods, salad dressings, and chocolates
